# Significantly Improved Cold Preservation of Rat Hind Limb Vascularized Composite Allografts Using the New PrC-210 Free Radical Scavenger

**DOI:** 10.3390/ijms25031609

**Published:** 2024-01-28

**Authors:** William E. Fahl, Zeeda H. Nkana, Maya M. Gitter, Weifeng Zeng, Aaron M. Dingle

**Affiliations:** 1 Wisconsin Institute of Medical Research, University of Wisconsin-Madison, 111 Highland Ave., Madison, WI 53705, USA; maya.coumbe18@gmail.com; 2 Division of Plastic Surgery, Department of Surgery, University of Wisconsin-Madison, 600 Highland Ave., Madison, WI 53705, USA; nkana@wisc.edu (Z.H.N.); wzeng28@wisc.edu (W.Z.); dingle@surgery.wisc.edu (A.M.D.)

**Keywords:** reactive oxygen species, cold-ischemia, ischemia-reperfusion

## Abstract

Vascularized composite allotransplantation (VCA) represents a promising reconstructive solution primarily conducted to improve quality of life. However, tissue damage caused by cold-ischemia (CI) storage prior to transplant represents a major factor limiting widespread application. This study investigates the addition of the novel free radical scavenger PrC-210 to UW Organ Preservation Solution (UW Solution) to suppress CI-induced skeletal muscle injury in a rat hind limb amputation model. Lewis rats received systemic perfusion of UW solution +/− PrC-210 (0 mM control, 10 mM, 20 mM, 30 mM, or 40 mM), followed by bilateral transfemoral amputation. Limbs were stored in 40 mL of the same perfusate at 4 °C for 48 h. Muscle punch biopsies were taken at set times over the 48 h cold-storage period and analyzed for caspase-3,7 activity, cytochrome C levels, and qualitative histology. A single 15 s perfusion of PrC-210-containing UW Solution conferred a dose-dependent reduction in CI-induced muscle cell death over 48 h. In the presence of PrC-210, muscle cell mitochondrial cytochrome C release was equivalent to 0 h controls, with profound reductions in the caspase-3,7 apoptotic marker that correlated with limb histology. PrC-210 conferred complete prevention of ROS-induced mitochondrial lysis in vitro, as measured by cytochrome C release. We conclude that the addition of 30 mM PrC210 to UW Solution conferred the most consistent reduction in CI limb damage, and it warrants further investigation for clinical application in the VCA setting.

## 1. Introduction

More than 200 patients have received vascularized composite allotransplantations (VCA), with the majority comprised of upper extremity and craniofacial transplantations [1]. There are an estimated 2.2 million individuals in the United States alone living with limb loss [2], and as an emerging field, VCA technology is yet to be widely applied in the clinical setting. Military personnel are at greater risk of traumatic limb and craniofacial injury than civilians [2,3,4]. Since the first successful hand transplant in 1998 and face transplant in 2005, VCA transplantation has emerged as a viable clinical reconstructive option for those who have survived catastrophic injuries, such as limb amputation or craniofacial disfigurement [4]. The emerging field of VCA has been enabled by advances in solid organ transplantation, microsurgery, and immunosuppression [5]. These advances allow for the transplantation of the multiple tissue types within a VCA as a single functional unit with the potential to outperform modern prosthetics [5] VCA represents a viable reconstructive option for individuals presenting with complex limb defects with distinct functional and cosmetic outcomes that are currently postulated to best improve quality of life (QOL) for bilateral amputees; however, it must be noted that current QOL measures may be insufficient [5,6]. Despite the benefits of VCA, issues stemming from tissue preservation techniques remain a significant limitation.

The current standard for VCA tissue preservation is static cold storage, which involves flushing the tissue with a preservation solution, most commonly UW Solution (ViaSpan), and then maintaining storage at 4 °C until transplantation; this storage constitutes cold-ischemia (CI) time. Several reports e.g., Refs. [7,8], have described the sources of damage during CI and subsequent warm reperfusion in transplantation:

1. Under hypoxic conditions, which include organ cold-ischemia (CI), the mitochondrial respiratory chain produces both nitric oxide (NO•) and reactive oxygen species (ROS), which both result in the toxic chemical modification of cellular nucleic acids, proteins, and lipids, and can include mitochondrial lysis;
2. Under oxidative conditions, which include organ warm-reperfusion, the mitochondrial respiratory chain, as well as other post-ischemia cell mechanisms, produce a bolus of ROS. This warm-reperfusion-associated ROS over-production results in the oxidation of cellular nucleic acids, proteins, lipids, glutathione, and more.

Prolonged CI storage induces tissue damage and chronic complications because of the biologically heterogeneous nature of tissues comprising a VCA graft, each of which has a varied degree of susceptibility to ischemia-induced injury [9,10,11]. It is generally accepted that skeletal muscle is the most vulnerable VCA tissue because of its high metabolic need and, hence, mitochondria content. Amputates containing more muscle (e.g., upper extremity amputation) are more susceptible to ischemic death, resulting in greater neuromuscular deficit post-transplant than that in amputates contacting less muscle (e.g., digital amputation) [12,13].

PrC-210 is a novel aminothiol ROS scavenger that can be administered orally, intravenously, and topically with no measurable limiting side effects [14]. Unlike antioxidants that act indirectly over a period of hours to days, PrC-210 directly scavenges ROS within seconds to minutes of administration to confer 100% protection. PrC-210 is currently the most efficient ROS scavenger in existence; it has demonstrated efficacy in acute murine renal and cardiac ischemia–reperfusion injury (IRI) models, with profound reductions in kidney capsule IRI and significant reductions in cardiac muscle death [15]. The present study was performed to determine the optimal PrC-210 concentration in UW solution, if any, that would reduce apoptotic cell death and prolong the preservation time of VCA grafts beyond what is currently possible with UW solution alone. As a first step to determine PrC-210 utility in supporting VCA transplants, we measured the ability of PrC-210 to reduce the degree of apoptosis and tissue decomposition in the skeletal muscle of amputated rat hind limbs preserved over a 48 h period.

## 2. Results

### 2.1. Limb Muscle Cell Death during Storage in Cold UW Solution

**Figure 1:**
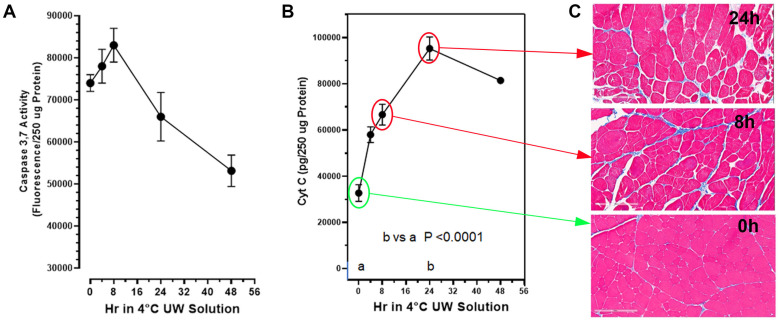
Expression of cell death biomarkers in amputated rat hind limbs perfused and stored in 4 °C UW Solution without PrC-210 over 48 h. Hind limbs were perfused in situ in rats with room-temperature UW Solution; the limbs were surgically removed and then stored in 4 °C UW Solution for the indicated times. Muscle biopsies were taken at indicated times for cell death biomarker assays and histology. (**A**) Caspase 3,7 activity in the muscle supernates was measured in a 60 min enzymatic assay as described in the Methods. Caspase-3,7 activity in the same muscle biopsy supernates significantly increased (*p* = 0.012) with 8 h storage over the zero-time muscle control caspase background. (**B**) Cytochrome C in muscle supernates was measured by ELISA as described in the Methods. A minimum of 5 muscle samples from 5 separate limbs were studied at each time point; both mean values and STDs are shown at each point released from its normal mitochondrial membrane location into the post-mitochondrial supernate, which increased by 80% at 4 h of storage and by over 300% (*p* < 0.0001) by 24 h of storage in UW Solution. Both the cytochrome C and caspase-3,7 activities were measured as surrogate markers of CI-induced muscle cell death. (**C**) Histology demonstrating degrading tissue quality over time (bottom to top) with the absence of PrC-210 over 24 h (Gomori’s trichrome, 20× magnification, scale bars = 200 µM). Histological presentations of tissue damage were found to correlate with cytochrome C cell death marker levels over time (**B**,**C**).

The extent of limb muscle cell death (apoptosis) during cold-ischemia (CI) storage, following the in situ perfusion of the limbs with UW Solution and then amputation, was measured with two cell death biomarkers in the tissue at multiple time points over 48 h of storage in 4 °C UW Solution (Figure 1). Free cytochrome C.

Muscle cell histology was acquired for the majority of the cold-storage rat limbs (Figure 1A,C). Muscle histology was qualitatively assessed using Gömöri trichrome stain, which highlights the extracellular matrix in blue and myocytes in pink.

Qualitative assessment demonstrated degenerating attachment of skeletal muscle to the extracellular matrix (white space between cells), as well as increasingly injured myocytes as a function of time (Figure 1C). Injured myocytes were characterized as irregular in shape, inconsistent cytoplasmic consistency in texture and/or color, and the presence of holes (white space within the cytoplasm). Figure 1B,C illustrate that the progressive degeneration of muscle cell histology correlated very well with the increase in the cytochrome C death marker in post-mitochondrial supernates of the same muscles, which reflects lysis of the muscle cell mitochondria.

### 2.2. PrC-210 Protection of Rat Limb Muscle Mitochondria

Muscle cell mitochondrial function affects both the (i) levels of reactive oxygen and nitric oxide species generated during CI and (ii) survival of muscle cells during CI. As a first step to determine whether PrC-210 would be protective to rat VCA limbs during their cold storage and follow-on transplant, we explored whether PrC-210 would significantly protect the rat limb muscle cell mitochondria in vitro when the mitochondria were exposed to a significant ROS insult, similar to that encountered by muscle cell mitochondria during CI-storage and post-transplant ischemia–reperfusion insults. If we observed PrC-210 suppression of an ROS insult in vitro, then it would provide rationale for using PrC-210 in next-step limb-transplant studies.

**Figure 2:**
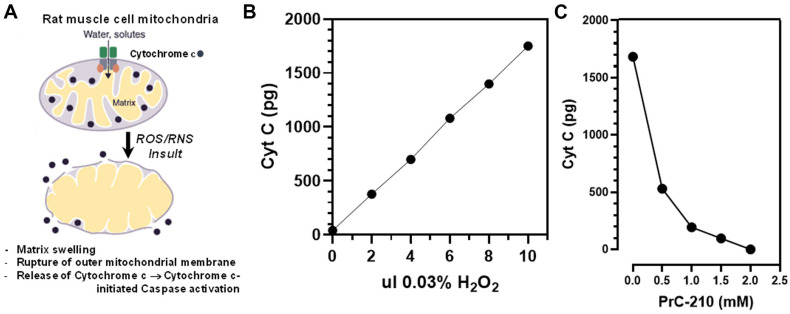
(**A**) Diagram of rat skeletal muscle cell mitochondrial structure showing the association of cytochrome C within the inner membrane scaffolding of the mitochondria. The insult and rupture of mitochondria releases cytochrome C, which was then measurable in post-mitochondrial supernates. (**B**) Aliquots of an untreated, rat muscle homogenate were exposed to a standard hydroxyl radical (•OH) generator (i.e., Fe^++^ + adenosine, plus increasing aliquots of added 0.03% H_2_O_2_ to initiate •OH production). Cytochrome C released from mitochondria (“free”) was quantified by ELISA. (**C**) PrC-210 dose-dependent suppression of “free” cytochrome C levels in muscle homogenate induced by •OH generator. PrC-210 was added to the muscle homogenate incubations at the indicated concentrations 10 min before the addition of 10 µL of 0.03% H_2_O_2_ to the homogenate and generator system. Following incubation (20 min, 37 °C), a 10 µL reaction aliquot was removed and assayed by ELISA for the free cytochrome C level.

To test PrC-210 protective efficacy, we (i) prepared a homogenate of normal rat hind limb muscle, (ii) put in place an •OH generator in in vitro incubations, (iii) quantified the •OH-induced mitochondrial damage using the cytochrome C biomarker, and (iv) determined the capacity of PrC-210 to protect the mitochondria at clinically relevant concentrations (Figure 2A–C) from this ROS insult.

Figure 2A illustrates the normal location of cytochrome C associated with the inner membranes of mitochondria. In Figure 2B, muscle homogenate incubated with an •OH generator, in this case FeCl_2_ plus ADP plus H_2_O_2_ in a Fenton reaction caused a linear increase in cytochrome C release from ruptured muscle cell mitochondria as the severity of the •OH insult increased. In Figure 2C, the addition of PrC-210 1 min before adding H_2_O_2_ to the incubations reduced the cytochrome C damage signal to the background level (at ~2 mM) in a PrC-210 concentration-dependent manner.

### 2.3. PrC-210 Reduction in CI-Induced Rat Muscle Cell Death in UW Solution Cold Storage

**Figure 3:**
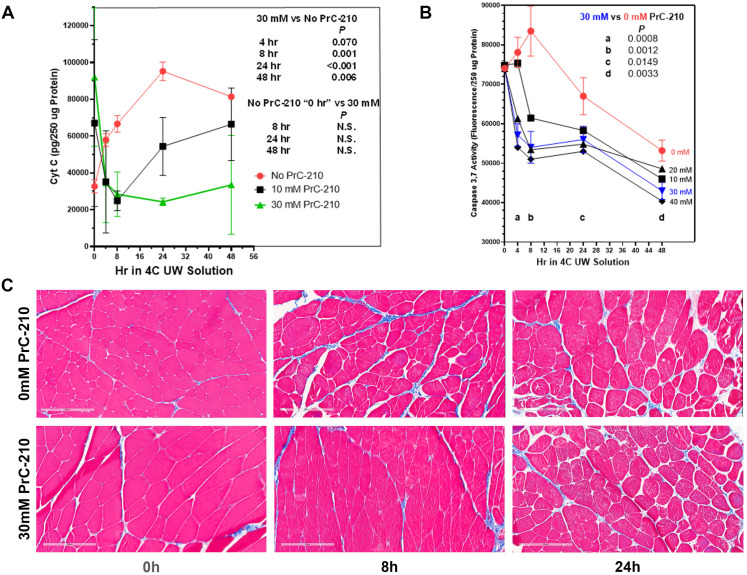
PrC-210 concentration-dependent suppression of rat limb muscle cell death during prolonged (48 h) amputated limb storage in 4 °C UW Solution. Rat hind limbs were perfused once, in situ, with room-temperature UW Solution containing the indicated PrC-210 concentration. Amputated limbs were then stored in 4 °C UW +/− PrC-210 for up to 48 h. Muscle punch biopsies were taken at indicated times and either (i) stored in liquid nitrogen (tissue post-mitochondrial supernates were then prepared and assayed for cell death biomarkers) or (ii) fixed in 10% formalin prior to histology workup and hematoxylin and eosin and Gömöri trichrome staining. Rat limb muscle post-mitochondrial supernate Cytochrome C (**A**), Caspase 3,7 (**B**), and trichrome histology (**C**) (scale bars = 200 µM) from the same limb muscles are shown.

To determine the ability of PrC-210 to reduce or eliminate the CI-induced muscle cell death observed in Figure 1, rat hind limbs were flushed in situ with PrC-210 augmented UW Solution. Figure 3A shows the considerable increase (red line) in muscle cell cytochrome C over 48 h in 4 °C UW Solution alone.

The addition of PrC-210 to UW Solution at 10 mM or 30 mM resulted in a clear concentration-dependent reduction in the Cytochrome C cell death marker. It is striking that, with 30 mM PrC-210 (green line) added, the Cytochrome C biomarker was reduced to the background level at every timepoint under 48 h of cold storage. With 10 mM PrC-210 (black line), protection was significant, but less so than that at 30 mM.

Figure 3B, likewise, shows for the Caspase 3,7 cell death marker that the addition of 30 mM PrC-210 significantly reduced the caspase at every time point throughout 48 h of 4 °C storage (*p* values 0.0149 through 0.0008). For other concentrations of PrC-210, there was generally a dose-dependent protection.

Figure 3C’s histology images clearly support the cytochrome C and caspase results; that is, the limb muscle at 8 h of cold storage appeared no different to the histology of the rat muscle immediately after its removal from the rat.

## 3. Discussion

The injury to VCA transplant tissues from cold-ischemia (CI) storage, when coupled with warm-reperfusion injury upon transplant, remains a significant problem [12,13,16]. As is the case with solid organ transplants, the combination of ischemia–reperfusion injury (IRI) manifests as eventual graft failure. Graft failure is a primary reason limiting further use and acceptance of VCA transplant [17]. Though cold storage of donor VCA tissue in UW Solution has the capacity to extend transit times, VCA graft failure clearly remains associated with extended cold-ischemia storage time. In this study, we sought to determine if PrC-210 would suppress, or prevent, damage to the skeletal muscle of rat limbs induced during the CI to which transplant organs are often exposed. Our data show that rat limb muscle cell death from extended cold-ischemia in the absence of PrC-210 is substantial, and the addition of PrC-210 to UW solution reduces CI-induced muscle apoptosis in a concentration-dependent manner. When measured by muscle cell mitochondrial cytochrome C release, CI-induced apoptosis in limbs protected by 30 mM PrC-210 is virtually eliminated for 48 h, i.e., it is equivalent to that of the control rat limbs at 0 h (Figure 3A). When measured by the caspase-3,7 death marker, the protective effect of 30 mM PrC-210 against CI-induced cell death is profound (Figure 3B). These data are supported by the histological evidence that control tissues stored in the absence of PrC-210 demonstrate morphological degradation at 8 h, while tissue preserved in 30 mM PrC-210 was morphologically equivalent to that at 0 h (Figure 3C). Furthermore, the tissue degradation observed in control tissues (0 mM PrC-210) directly correlated with cell death quantified using cytochrome C release (Figure 1). PrC-210 conferred complete prevention of ROS-induced rat muscle mitochondrial lysis, as measured from rat muscle in vitro by cytochrome C release (Figure 2C). Complete protection was conferred at a PrC-210 concentration of 2 mM, a concentration known to be achieved and tolerated in the plasma of PrC-210-protected animals.

While VCA is still in its infancy, it has proven to be a promising reconstructive technique, exerting positive impacts on patient lives that are at least comparable to those of modern prosthetic alternatives [5]. Despite the combined aesthetic and functional advantage of VCA, widespread clinical application is limited in part by high rates of rejection and logistical issues conferred by preservation and transplantation [18,19]. One of the primary elements that limits tissue survival is ischemic injury of the high-metabolic-activity tissues, particularly skeletal muscle, comprising VCAs; this represents a significant barrier to advancing the field [13].

The rat muscle cell death corresponding to extended cold-ischemia storage in the absence of PrC-210 is substantial. At 24 h of storage, there was a 300% increase (*p* < 0.0001) in the muscle cell cytochrome C damage marker, which indicates substantial muscle cell mitochondria lysis (Figure 1B). The cytochrome C damage marker was mirrored by both the (i) increase in the muscle caspase-3,7 death/apoptosis marker, as well as (ii) the deterioration in muscle cell histology (Figure 1). After a significant increase in caspase-3,7 (*p* = 0.012) at 8 h of CI, the reduced caspase was a function of this protease’s auto-digestion and muscle cell lysis. Poyton [7] and Castello [8] attribute this organ CI damage to NO and ROS generated during the hypoxia of cold storage. The ROS-scavenging agent, PrC-210, has previously been shown to reduce both of these insults [20] and is therefore expected to confer a substantial improvement in the VCA transplant process.

The capacity to significantly suppress both CI and warm-reperfusion injury would be expected to (i) increase the donor pool of available organs and (ii) reduce the incidence of VCA graft failure, as well as any associated costs, such as the need for, e.g., revision surgery or extended hospitalization.

Disruption to normal mitochondrial oxidative function through insults, like CI, has the capacity to determine how oxygen and its free-radical forms cause cell damage. Mitochondrial oxidative function is particularly critical to limb skeletal muscle cells because of their: (i) high mitochondrial content and (ii) required ATP energy output to service the functional muscle cells. It is, therefore, highly significant that PrC-210 suppressed, to background, both (i) cytochrome C release in intact rat limbs stored at 4 °C for 48 h (Figure 3A, *p* = N.S.) and (ii) cytochrome C release from rat muscle mitochondria in vitro (Figure 2C) that were exposed to a direct ROS insult. Furthermore, the protection from apoptosis attributed to PrC-210 was achieved at a concentration (2 mM) previously shown to be readily achieved in the plasma of rats and mice with no detectable toxicities.

An important advantage of PrC-210 is its “immediate action” as an ROS scavenger [20]. Its capacity for immediate action is attributed to PrC-210’s small size (MW: 148) which allows simple transmembrane diffusion, while (+) charges function to localize it around (−) charged nucleic acids and proteins in both mitochondria and nuclei. These characteristics of PrC-210 explain its efficiency for capturing the •OH produced in cells [21]. In its active thiol form, at pH 7.2, PrC-210 has a half-life of 3.5 h in rat plasma. Perfusion at 0 h with 30 mM PrC-210 in UW Solution (Figure 3A) suppressed the cytochrome C signal to the background level over 48 h of 4 °C storage. This indicates that ~4–5 half-lives at pH ~7.2 still provide a PrC-210 thiol concentration within the muscle cell milieu that protects the integrity of the mitochondrial and nuclear compartments.

Finally, the ability of PrC-210 to suppress skeletal muscle damage in amputated limbs from prolonged (48 h) CI insult (Figure 3A) contributes to a growing body of evidence supporting the use of PrC-210 to suppress ischemia–reperfusion injury in a range of clinically relevant scenarios [15,20]. Owing to its small molecular size and solubility, PrC-210 is likely to be applicable to any clinical scenario where blood flow is stopped and restarted.

Our data demonstrate that: (i) rat limb muscle cell death from extended CI storage (up to 48 h) in the absence of PrC-210 is substantial; (ii) the addition of PrC-210 to UW Solution confers a dose-dependent reduction in CI-induced muscle cell death over 48 h, which, when measured by muscle cell mitochondrial cytochrome C release, is equivalent to control rat limbs at 0 h, and when measured by the caspase-3,7 death marker or direct muscle histology, is profound; and, (iii) in in vitro rat limb muscle assays, PrC-210 conferred complete prevention of ROS-induced mitochondrial lysis, as measured by cytochrome C release, at a PrC-210 concentration of 2 mM. These findings indicate that 30 mM of PrC-210 is best suited to improve the preservation of skeletal muscle in VCAs over extended periods. Future work will address the capacity of PrC-210 to (1) enhance the preservation of, and (2) reduce reperfusion injury to, VCAs in vivo in both rodents and a clinically translatable swine model.

## 4. Materials and Methods

### 4.1. Animals

Lewis rats (male; 300–450 g) were purchased from Charles River (Wilmington, MA, USA) and housed in the University of Wisconsin Laboratory Animal Facility. This research was prospectively approved by the University of Wisconsin School of Medicine and Public Health’s Institutional Animal Care and Use Committee (IACUC) (Protocol: M006347) and the United States Department of Defense’s Animal Care and Use Review Office (ACURO). All animal care and procedures were performed in accordance with guidelines outlined by the IACUC and ACURO.

### 4.2. Materials

The synthesis of the novel aminothiol, PrC-210 HCl, has been previously described [14]. PrC-210 crystals were dissolved directly (instantly) into UW Solution to achieve the desired PrC-210 concentration; 5N NaOH was added to re-achieve the unmodified UW Solution pH of 7.4 (i.e., 0.0619 μL of 5N NaOH per μmol of PrC-210 HCl salt added to UW solution). All other materials were obtained from Sigma Aldrich (St. Louis, MO, USA), Thermo Fisher Scientific (Waltham, MA, USA), and Promega Corporation (Madison, WI, USA).

### 4.3. Experimental and Surgical Procedure

Lewis rats were anesthetized using 3% isoflurane, followed by exposure of the pericardial cavity for systemic perfusion. Systemic perfusion was utilized to ensure simultaneous initiation of ischemia with equal exposure to preservation solutions prior to amputation. The ascending aorta was catheterized via the left ventricle, with an additional incision made in the right atrium for drainage of the perfusate [22]. The perfusate was either UW Solution alone (0 mM PrC-210, control) or UW solution with dissolved PrC-210 to achieve concentrations of 10 mM, 20 mM, 30 mM, and 40 mM. The total volume of full-body cardiac perfusion was estimated to be 2 times the total body blood volume (i.e., 6% of the total body weight, with 5 units of heparin per mL of UW solution with or without PrC-210). Following full-body cardiac perfusion at room temperature, both hind limbs underwent transfemoral amputation, were placed into 40 mL of UW solution containing the same PrC-210 concentration as the perfusate, and stored at 4 °C for 48 h. Two punch biopsies (5 mm, Cen-Med Enterprises, New Brunswick, NJ, USA) per limb were taken at 0, 4, 8, 24, and 48 h post-amputation. At each time point, one biopsy was immediately frozen in liquid nitrogen and stored at −80 °C for the biomarker assay, and one biopsy was fixed in 10% neutral buffered formalin and stored at 4 °C for histological examination.

### 4.4. Hind Limb Muscle Caspase 3,7 Assays

Activated caspase-3 and 7 activity in hind limb homogenate supernates was determined using the ApoONE fluorescent substrate (Promega, Madison, WI, USA). Briefly, individual, thawed muscle biopsies (80–100 mg biopsy) were mixed with 1.0 mL of 4 °C lysis buffer containing 50 mmol/L Na HEPES, pH 7.4, 100 mmol/L NaCl, 1 mmol/L ethylene diamine tetra-acetic acid, and 10% glycerol and homogenized at 4 °C for 30 s with an Omni tissue homogenizer. The muscle biopsy homogenates were centrifuged at 14,000× *g* (4 °C) in an Eppendorf microfuge for 20 min. The post-mitochondrial supernates were assayed for cytochrome C and caspase-3,7 activity, and the protein content was determined by the Bradford method using bovine serum albumin (BSA) as the standard. The activated caspase-3,7 assay was performed as follows: 50 μL of muscle supernate (≈150 μg of supernate protein) was mixed with 50 μL of the Apo-ONE substrate solution in the wells of a black, opaque, 96-well plate to initiate the 60 min reaction. The plates were shaken at 200 RPM at 37 °C for 60 min. The DEVD (Asp-Glu-Val-Asp peptide) caspase substrate peptide cleavage was measured using a Clariostar fluorescent plate reader (BMG Labtech, Cary, NC, USA) at an excitation wavelength of 499 nm and an emission wavelength of 521 nm. A caspase internal standard was included in each experiment.

### 4.5. Hind Limb Muscle Cytochrome C Assays

The levels of free cytochrome C were determined by ELISA (abcam # 210575). Briefly, dilutions of post-mitochondrial hind limb muscle supernates were added to antibody-precoated plates and incubated for 1 h at 37 °C. Following secondary antibody incubation, the plate was washed; avidin-conjugated horseradish peroxidase was added, followed by washing and TMB substrate addition. The reaction was incubated for 10 min, stopped with sulfuric acid, and read at 450 nm.

### 4.6. Histological Assessment of Hind Limbs

The hind limb muscle biopsy samples were fixed in 10% neutral buffered formalin and embedded in paraffin; sections were then mounted and stained with hematoxylin and eosin (H&E) and Gömöri trichrome. Slides were scanned using a 20× objective in an Aperio Digital Pathology Slide Scanner and viewed using Aperio ImageScope (v.12.3.3.5048). Qualitative assessment was performed on each biopsy at low (×4) and high (×20) magnifications, focusing on the basic assessments of myocyte injury. Injured myocytes were characterized as being irregular in shape, inconsistent cytoplasmic consistency in texture and/or color, and the presence of holes (white space within the cytoplasm).

### 4.7. Rat Muscle Cell Mitochondria

A five-gram sample of rat hind limb muscle was homogenized at 4 °C in five volumes of 0.15 M Tris HCl buffer, pH 7.4. To determine whether the addition of exogenous PrC-210 suppressed mitochondrial damage from an •OH generator and the accompanying release of the cytochrome C biomarker, in a 76 µL reaction volume (in a 0.5 mL Eppendorf tube), we added: 44 µL of muscle homogenate, 10 µL of 0.15 M Tris (pH 7.4), 10 µL of PrC-210 dilution or water (PrC-210 was added 10 min before the Fe^++^ + ADP + H_2_O_2_ •OH generator), 6 µL of FeCl_2_ (6 mM; FW:127) and adenosine 5′-diphosphate sodium salt (24 mM; FW: 427), and 10 µL of 0.03% H_2_O_2_ [23]. After shaking for 10 min at 37 °C, a 10 µL reaction aliquot was removed, and this was used to quantify free cytochrome C by the ELISA assay described above.

### 4.8. Statistics

Data are expressed as means +/− STDs. One-tailed Student *t*-tests were used to determine statistical difference and *p* values using GraphPad Prism 7.03 software. *p* values < 0.05 were considered significant.

## Data Availability

All materials are available by request to the corresponding author.
